# Test of a workforce development intervention to expand opioid use disorder treatment pharmacotherapy prescribers: protocol for a cluster randomized trial

**DOI:** 10.1186/s13012-017-0665-x

**Published:** 2017-11-15

**Authors:** Todd Molfenter, Hannah K. Knudsen, Randy Brown, Nora Jacobson, Julie Horst, Mark Van Etten, Jee-Seon Kim, Eric Haram, Elizabeth Collier, Sanford Starr, Alexander Toy, Lynn Madden

**Affiliations:** 1 Department of Industrial and Systems Engineering, University of Wisconsin-Madison, 1513 University Ave., Madison, WI 53706 USA; 2 Department of Family Medicine and Community Health, University of Wisconsin-Madison, 1100 Delaplaine Ct., Madison, WI 53715-1896 USA; 3 Institute for Clinical and Translational Research, University of Wisconsin-Madison, 4116 Signe Skott Cooper Hall, 701 Highland Ave, Madison, WI 53705 USA; 4 Department of Behavioral Science and Center on Drug and Alcohol Research, University of Kentucky, 845 Angliana Ave., Room 204, Lexington, KY 40508 USA; 5 1075E Educational Sciences, 1025 West Johnson St., Madison, WI 53706-1706 USA; 6 1 West Wilson St. Rm 850, Madison, WI 53703 USA; 7 30 E. Broad St., 8th Floor, Columbus, OH 43215 USA

**Keywords:** Evidence-based practice implementation, Medication-Assisted Treatment, Buprenorphine, Naltrexone, Addiction treatment

## Abstract

**Background:**

Overdoses due to non-medical use of prescription opioids and other opiates have become the leading cause of accidental deaths in the USA. Buprenorphine and extended-release naltrexone are key evidence-based pharmacotherapies available to addiction treatment providers to address opioid use disorder (OUD) and prevent overdose deaths. Treatment organizations’ efforts to provide these pharmacotherapies have, however, been stymied by limited success in recruiting providers (physicians, nurse practitioners, and physician assistants) to prescribe these medications. Historically, the addiction treatment field has not attracted physicians, and many barriers to implementing OUD pharmacotherapy exist, ranging from lack of confidence in treating OUD patients to concerns regarding reimbursement. Throughout the USA, the prevalence of OUD far exceeds the capacity of the OUD pharmacotherapy treatment system. Poor access to OUD pharmacotherapy prescribers has become a workforce development need for the addiction treatment field and a significant health issue.

**Methods:**

This cluster randomized controlled trial (RCT) is designed to increase buprenorphine and extended-release naltrexone treatment capacity for OUD. The implementation intervention to be tested is a bundle of OUD pharmacotherapy capacity building practices called the Prescriber Recruitment Bundle (PRB), which was developed and piloted in a previous statewide buprenorphine implementation study. For this cluster RCT, organizational sites will be recruited and then randomized into one of two arms: (1) control, with treatment as usual and access to a website with PRB resources, or (2) intervention, with organizations implementing the PRB using the Network for the Improvement of Addiction Treatment organizational change model over a 24-month intervention period and a 10-month sustainability period. The primary treatment outcomes for each organizational site are self-reported monthly counts of buprenorphine slots, extended-release naltrexone capacity, number of buprenorphine patients, and number of extended-release naltrexone patients.

This trial will be conducted in Florida, Ohio, and Wisconsin, resulting in 35 sites in each arm, for a total sample size of 70 organizations.

**Discussion:**

This study addresses three issues of substantial public health significance: (1) the pressing opioid misuse epidemic, (2) the low uptake of OUD treatment pharmacotherapies, and (3) the need to increase prescriber participation in the addiction treatment workforce.

**Trial registration:**

ClinicalTrials.gov NCT02926482,

## Background

Overdoses due to non-medical use of prescription opioids and other opiates have become the leading cause of accidental deaths in the USA [1]. Opioid use disorder (OUD) pharmacotherapies are a key evidence-based intervention available to specialty addiction treatment organizations to address the increase in OUDs and reduce overdose deaths [2–4]. This study focuses on two of these pharmacotherapies, buprenorphine and extended-release naltrexone. Buprenorphine is an opioid agonist therapy that binds to the opioid receptor to reduce the effects of opioids. Buprenorphine enhances retention in treatment and reduces self-reported use of opioids, criminal activity, and mortality [3, 5, 6]. Buprenorphine can only be prescribed by health care providers who have completed training to obtain a waiver to treat a limited number of patients [7]. Patients take the medication daily via tablet or film formulations; a longer-acting implant formulation is also available. Extended-release naltrexone (Vivitrol®) is an opioid antagonist medication that blocks the opioid receptor and also has improved retention rates when compared to placebo [4, 8]. Extended-release naltrexone is typically injected once a month at prescriber’s office location. While these pharmacotherapies hold great promise, they are underutilized in OUD treatment. Of the 2.5 million Americans 12 years of age or older with OUDs [9], fewer than 128,000 of those attending specialty treatment programs had treatment plans that included pharmacotherapy [10].

Treatment organizations’ efforts to provide OUD pharmacotherapies have been hindered by limited success in recruiting providers to prescribe these medications. In previous studies [11], physician prescribers have expressed hesitancy to prescribe OUD treatment pharmacotherapies due to feeling that treating OUD patients can be difficult, limited confidence in treating OUD patients, lack of institutional support, inadequately trained staff, time constraints, poor reimbursement rates, and regulatory barriers to OUD pharmacotherapy [11–15]. One regulatory barrier specific to buprenorphine requires prescribers to apply for a waiver that limits them to treating 30 patient slots in year 1, 100 slots in year 2, and up to 275 slots per year thereafter. Legislation passed in 2016 expands prescribing privileges to advanced practice registered nurses and physician assistants, who must also complete training and obtain a waiver to treat a limited number of patients [16].

These conditions have created significant barriers to OUD treatment capacity and implementation of these pharmacotherapies. A recent analysis found that 24.1% of specialty treatment organizations using buprenorphine pharmacotherapy had to turn patients away due to insufficient prescribing capacity [17]. About 43% of the US counties have no buprenorphine prescribers; among states and the District of Columbia, 96% had OUD rates that exceeded their buprenorphine capacity [18].

Poor access to prescribers of OUD pharmacotherapy has become a workforce development need in the addiction treatment field. Traditionally, organizations providing addiction treatment services have not developed competency in recruiting prescribers; rather, they have primarily relied on a clinical workforce of counselors. That is quickly changing, as addiction treatment providers are now being called upon to expand their clinical services into physician-supported areas such as primary care, mental health/psychiatry, and pharmacotherapy for addiction disorders. This study protocol’s aim is to address this emerging need by testing a bundle of practices, the Prescriber Recruitment Bundle (PRB), coupled with the Network for the Improvement of Addiction Treatment (NIATx) organizational change model to increase OUD treatment pharmacotherapy workforce capacity.

The PRB was developed to address the leading barrier to buprenorphine implementation, lack of physician prescribing capacity, as identified by 40 treatment organizations participating in the Ohio project titled “To Test a Payer/Treatment Agency Intervention to Increase Use of Buprenorphine.” This was a surprising finding because lack of reimbursement for buprenorphine was expected to be the most frequent and challenging barrier to expanding buprenorphine use. However, as the project proceeded and reimbursement barriers were removed, the lack of physicians to prescribe buprenorphine prevented its use within treatment organizations. As a result, different interventions began to be tested to increase buprenorphine prescriber capacity. The successful interventions were packaged into what became the PRB and later were tested by eight organizations in the project. The eight organizations that applied the PRB increased buprenorphine prescribing slots by 48.3–100%. The slots, once acquired, were mostly filled due to the pressing need for medication-assisted treatment services. The PRB pilot recruited 10 new physicians and increased buprenorphine prescribing slots across the eight organizations by more than twofold. The PRB developed and piloted was limited to a pre/post-evaluation on a convenience sample; this protocol will describe a randomized test of the PRB.

### Objectives

In this cluster randomized controlled trial (RCT), the primary aim is to test the impact of the PRB implemented in conjunction with the NIATx organizational change model, relative to the control, on (a) increasing the number of buprenorphine treatment slots and extended-release naltrexone capacity and (b) increasing the number of patients receiving buprenorphine and extended-release naltrexone in the participating addiction treatment organizations. A secondary aim is to test whether the PRB affects factors likely to be associated with prescriber recruitment, including resources dedicated to prescriber recruitment and physicians’ job satisfaction [19–21], and subsequently, whether these factors mediate the effects of the PRB on key outcomes. Lastly, qualitative methods will be used to study the context and processes that influence PRB adoption and fidelity, to develop a deeper understanding of how the PRB influences recruitment beyond the quantitative study variables, and to gain knowledge of what organizational processes contribute to successful licensed prescriber recruitment.

## Methods

### Trial design

In the intervention arm of this cluster randomized trial, the PRB will be implemented during a 24-month trial using the evidence-based NIATx organizational change model developed by our research center. The control arm will only receive access to the PRB materials online via a secure website. Organizations in the control arm will complete all the same data collection instruments as those in the intervention arm.

### Participants

We selected the three states (FL, OH, and WI) due to the states’ varied frequency of opioid analgesic prescribing and drug-poisoning deaths [22, 23]. These states offered a mix of urban and rural settings, presence of minority populations, strength of data collection and reporting systems, and representation of both Medicaid expansion and non-expansion states.

**Fig. 1:**
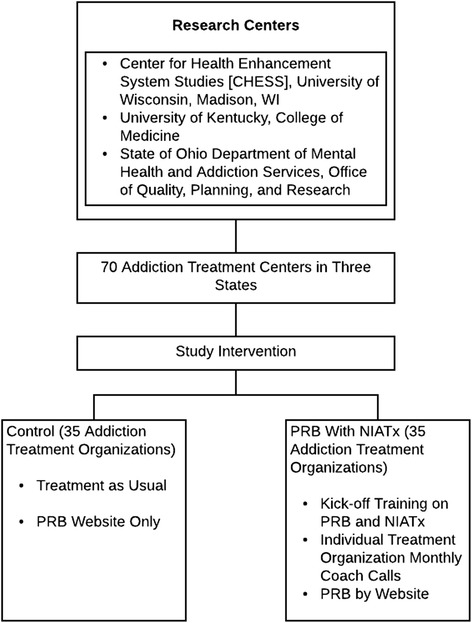
Consort diagram. *PRB* Prescriber Recruitment Bundle

In all three states, recruitment began with surveying all publicly funded organizations that are licensed to provide addiction treatment services and have at least 100 admissions per year. The state-based addiction treatment authorities provided a list of the organizations meeting these eligibility criteria. The survey assessed the organizations’ interest in increasing their buprenorphine prescribing capacity. Organizations expressing the need for greater OUD pharmacotherapy capacity were recruited into the trial through an invitation from the study team. Of 125 organizations indicating need for greater OUD pharmacotherapy, 24 organizations from Florida, 23 from Ohio, and 23 from Wisconsin agreed to participate, for a total of 70 organizational sites (Fig. 1, consort diagram). Thirty-five were placed in the intervention arm, and 35 were in the control arm.

### Randomization and consent procedures

The first stage of the block randomization within each state is to develop sampling matched organizations from those that agreed to participate, based on their current use of PRB practices (i.e., number of PRB practices being used 0–7) and whether they were currently prescribing buprenorphine (i.e., yes or no). As a result, 11–13 matched pairs are identified per state. For Ohio and Wisconsin, one matched pair will have two intervention sites to one control site, due to the overall uneven number (*n* = 23). A random number generator is utilized to assign organizations within each pair into one of the two study conditions. Following randomization, study staff will provide University of Wisconsin Institutional Review Board approved study information sheets to staff participants. No visible concealment will be applied. Blinding of participants and researchers is considered logistically infeasible.

### Interventions

The PRB uses two approaches to implementation: the NIATx organizational change model within a multi-organizational learning collaborative in the intervention arm and online PRB resources in both study arms. The PRB includes:

- Candidate identification strategies for finding prescribers interested in prescribing OUD pharmacotherapies. The Ohio “To Test a Payer/Treatment Agency Intervention to Increase Use of Buprenorphine” found the following physician specialties to be more likely to become opioid pharmacotherapy prescribers: (a) addiction medicine specialists, (b) psychiatrists who are already practicing within the organization, and (c) family medicine and internists in the community. Similarly, Rosenblatt et al. [24] observed that 81.9% of physicians with a Drug Enforcement Administration (DEA) waiver to prescribe buprenorphine came from a combination of the psychiatry, primary care, and addiction medicine specialties. The bundle recommends seeking physician prescribers from these specialties and to be aware that as of 2016, advanced practice nurses and physician assistants can also now prescribe buprenorphine [25].
- Prescriber education forum slide deck that can be used to inform prescriber groups and stimulate interest in OUD pharmacotherapy prescribing. Potential prescribers are often unaware of the practice opportunities in addiction medicine. Once they are aware of those opportunities, they are more likely to provide care to patients with addiction disorders [26]. Education forums can describe practice opportunities in the addiction field, how physicians and other clinicians can reduce the impact of OUDs, and give medical professionals the opportunity to express their interest in becoming OUD pharmacotherapy prescribers. The slide deck is intended for use by agency staff
- Prescriber-friendly workflow and risk-reduction strategies that provide clinical supports to assist prescribers with managing OUD pharmacotherapy patients. Two concerns of potential prescribers are that patients on opioid treatment pharmacotherapies require added effort and that these patients present additional clinical risk [27]. LaBelle et al. [28] reported that the use of nurses to assist physicians with the provision of buprenorphine treatment has led to expanded use of this pharmacotherapy. This strategy reduces the clinical workload on prescribers and allows for the use of a risk management plan based on existing clinical guidelines that also prevents the non-medical use or diversion of buprenorphine prescriptions [29]. Education can also be provided on how buprenorphine side effects and mortality are low, and side effects are less severe than other comparable opioid treatment alternatives [3].
- Academic detailing to recruit OUD prescribers. Academic detailing is an evidence-based tool to influence prescriber decision making and to recruit OUD prescribers. Developed by Soumerai et al. [30], academic detailing uses persuasive communication to influence physician behavior [31, 32]. This approach applied to recruiting OUD pharmacotherapy prescribers includes the following steps: (1) conducting interviews to investigate baseline knowledge of and motivations for buprenorphine treatment, (2) defining clear educational behavioral objectives, (3) establishing credibility by citing authoritative and unbiased sources of information and presenting both sides of controversial issues, (4) stimulating active physician and clinician participation in educational interactions, (5) using concise graphic educational materials, (6) highlighting and repeating the essential messages, and (7) providing positive reinforcement during follow-up visits. Agency staff are encouraged to use academic detailing strategies as part of physician recruitment.
- Telemedicine to provide access to OUD pharmacotherapies. Barriers of time and distance can limit access to OUD prescribers [11]. Buprenorphine prescribing physicians are often clustered in urban centers [24]. Some behavioral health organizations have begun to use telemedicine to increase patient access to services that are not available or if more capacity is needed in a given clinic [33–35]. For buprenorphine care, a buprenorphine prescriber can use videoconferencing to treat a patient at a remote location. Resources will be available in the PRB explaining how to use telemedicine for buprenorphine care to increase access to care and patient convenience.
- Promoting organizational leadership and culture to support the building of OUD pharmacotherapy capacity. Leadership and culture that supports use of opioid treatment pharmacotherapies, and the recruitment of OUD pharmacotherapy prescribers facilitates provision of this evidence-based practice [36]. Specific actions leaders can take to support recruitment include providing resources for recruiting and creating an attractive environment for applicants [37]. Examples of how to apply leadership skills to promote medication-assisted treatment use will be also included in the PRB.

**Fig. 2:**
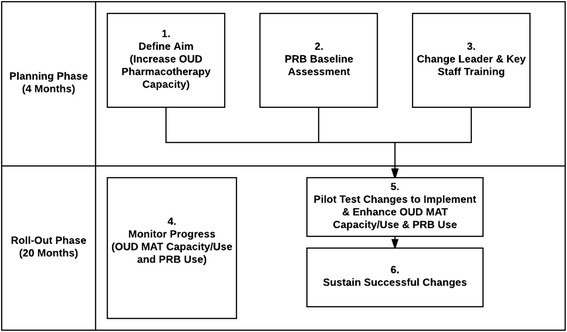
PRB implementation sequence (during the 24-month intervention period). *OUD* opioid use disorders, *PRB* Prescriber Recruitment Bundle, *MAT* Medication-Assisted Therapy

In the intervention arm, the PRB implementation will begin with a 4-month planning phase to identify practices that are not present or underutilized in the study sites. They will then be implemented in the rollout phase (Fig. 2). Within each treatment organization, we will engage management to participate in PRB practices: a top leader (the executive sponsor), the primary physician recruiter for the organization (the change leader) if different from the executive sponsor, and the clinical director.

During the planning phase, key components of the PRB implementation process (designed based on the evidence-based NIATx organizational change model [38, 39] include (1) defining the aim through a briefing between the executive sponsor and a project coach provided by the research team, (2) assessing PRB baseline to measure level of use of the different PRB practices and document the existing buprenorphine slots availability and extended-release naltrexone capacity, and (3) training leadership and prescriber recruiters to develop and implement an organizational change or a new practice from the bundle. The training will include all organizations within the state that were randomized to the intervention arm to encourage cross-organizational sharing. After the planning phase, a 20-month rollout phase occurs that includes (4) monitoring progress in increasing the number of buprenorphine slots, extended-release naltrexone capacity, and use of the PRB; (5) pilot testing of changes to implement and enhance PRB implementation using the evidence-based Plan-Do-Check (or measure)-Act approach [40]; and (6) sustaining successful changes by implementing a plan to institutionalize gains and avoid reverting to the old system.

Throughout the planning and rollout phases, each organization will have a coach. The coach is an expert in prescriber recruitment, OUD treatment, and organizational change who supports treatment organizations as they make, sustain, and spread PRB practices. Coaches help organizations think through key issues, offer process improvement training, and suggest changes during monthly coaching calls and e-mails as needed.

### Timeline

During project months 1 to 18, the study team will design the PRB intervention, complete IRB approval, and identify and recruit treatment organizations. In months 14 to 18, the study team will collect baseline data on number of assigned and open buprenorphine slots and extended-release naltrexone capacity. In months 15 to 42, the study team will implement the study arm interventions and collect outcome data on the 24-month staggered start intervention. During months 37 to 52, the study team will collect quantitative data on the sustainability of the changes. Data analysis, publication development, and dissemination of findings will occur at several points, but primarily during months 53 to 60.

### Sample size

In determining sample size, we fit a linear mixed-effects model to the monthly results for assigned and available buprenorphine slots to estimate the “PRB with NIATx organizational change model” effect. The power of the study design was determined by the anticipated standardized effect size based on the effects experienced in the PRB pilot study. The PRB pilot study, which included 8 organizations, found that PRB increased physician recruitment by 43.4–100%, which can be transformed into Cohen’s *d* = .394 on average [41]. Intraclass correlation (ICC) among sites affects the power of cluster randomized trials [42]. The estimate of ICC was around 0.10. We will have recruited 35 treatment sites and 35 comparison sites. Based on our previous and current projects with similar participants, we expect about 15% attrition by the end of the data collection, so we used a more conservative 20% attrition rate for our power calculations. With 28 organizations in each condition with an average of 150 buprenorphine slots in each organization, the study will achieve a power of .93 (*d* = .30) or higher (*d* > .30) with a type I error rate of 0.05. Power calculation for the cluster RCT was performed using Optimal Design Software [43].

### Data collection and measures

The primary outcome variables are the monthly number of (a) assigned and (b) open buprenorphine slots as well as (c) extended-release naltrexone capacity and (d) administrations over the past month. *Assigned slots* are defined as the number buprenorphine slots all prescribers affiliated with the organization have dedicated to the organization. For the buprenorphine data, on the first working Monday of the month, each organization will count the numbers of assigned and open buprenorphine slots. For extended-release naltrexone data, the number of extended-release naltrexone administrations for the previous month will be counted. These data will be collected during the 4-month planning phase, the 20-month rollout phase, and the 10-month sustainment phase, for a total of 34 months.

For the mediational analysis, at organizational baseline, mid-intervention, and the end of the intervention phases, the PRB change leader (or primary contact) will complete the organizational surveys for the PRB implementation fidelity. In addition, any physician affiliated with the organization (by employment or contract) will complete the Physician Worklife Survey [21].

The qualitative portion of the study will support the research questions by exploring facilitators and barriers to successful prescriber recruitment, as well as the process of PRB implementation within organizations and fidelity to PRB implementation sequence. Sampling for the qualitative component will promote integration of qualitative and quantitative approaches by drawing upon findings from the quantitative assessments. Semi-structured interviews will be conducted with up to 15 organizations in the intervention arm that have had success at implementing the PRB (at least 5 of the 7 practices from the PRB fidelity scale) and up to 15 organizations that have implemented fewer than 3 PRB practices at the end of the first year or mid-point of the intervention. In this longitudinal design, selected organizations will be interviewed at end of the mid-point and at the end of the intervention period to allow for more in-depth exploration of what occurred within these organizations over time.

In the interviews, the closed-ended questions will be based on domains of the Burke-Litwin Model of Organizational Change: (1) leadership, (2) recruiting practices, (3) work-unit change, and (4) organizational motivational for recruitment [44]. The open-ended questions will allow for discovery of other factors affecting PRB implementation and prescriber recruitment.

### Data analysis

We intend to use an optimal design in which treatment arms are allocated following a multi-site cluster randomization procedure [43]. A preliminary analysis will compare baseline characteristics between intervention and control condition sites, as well as sites enrolled in the project and eligible sites that chose to not participate. Chi-square tests and linear models will be used to formally test for statistically significant baseline differences between the arms. Descriptive statistics and plots will be used to summarize the distribution of each of the analytic variables collected at each time point for each arm. The organizational site is the unit of analysis.

For the primary outcome analysis, mixed-effects models (random effects due to organization and fixed effects due to study arm and time) will be used for data analysis of the monthly number of assigned and open buprenorphine slots per organization, estimated extended-release naltrexone capacity, buprenorphine use, and extended-release naltrexone use. A number of organizational characteristics found by Knudsen et al. [45] to affect the presence of physicians in addiction treatment setting, including facility type, services offered, and organizational size, will be included in the models as covariates to properly and efficiently estimate the effect of PRB. We will examine the PRB effect at each time point using cross-sectional multilevel models and will also implement growth curve models across time that include the PRB effect, time, and PRB*time, as well as time-varying covariates and organizational characteristics. The available and open buprenorphine slots, extended-release naltrexone capacity, buprenorphine use, and extended-release naltrexone use will be measured repeatedly for the same subjects, and it is expected that these values will be correlated over time. Therefore, instead of assuming an independence (i.e., zero correlation) or a compound symmetry covariance structure (i.e., a constant correlation regardless of the proximity of measurement time points), we will allow errors to be auto-correlated or Toeplitz structured, denoted as AR(p) or TOEP(p), in the growth curve models. This can be done by allowing additional parameters in the mixed-effects model error structures that represent the correlation between the adjacent measures of outcomes, and is reduced as the measures become further apart [46]. For the meditational analysis, we will examine the mediating effects of resources dedicated to physician satisfaction and PRB implementation fidelity through a causal mediational analysis. Each of the factors will be aggregated at the organizational site level and measured using mixed-effects models. Through a mediation analysis [47, 48], we can estimate the direct (buprenorphine slots, extended-release naltrexone, buprenorphine use, and extended-release naltrexone use with and without PRB) and indirect effects (resources dedicated to prescriber recruitment, staff prescriber satisfaction, and PRB implementation fidelity) of the PRB on the outcome measures. Using the R package “mediation,” we will estimate the causal mediation effects, examine moderated mediation effects, and conduct sensitivity analysis at each time point as well as across time [49].

The qualitative analysis will include deductive and inductive components. The Burke-Litwin model will provide the domains for a directed content analysis [50]. An inductive approach based on grounded theory and dimensional analysis [51–54] will be used to identify and explore additional contextual and processual factors that affect PRB implementation. Both directed and inductive analyses will include within-site and cross-site comparisons. Qualitative analysis will begin with the first interview and will continue in tandem with data collection, allowing the investigators to use later interviews to delve into factors identified in early interviews. Together, these approaches will produce a thorough description of PRB implementation that will be combined with the quantitative results to deepen our understanding of how the PRB works, promoting insights that can be applied to future dissemination of the PRB, and to discover strategies beyond the PRB that contributed to prescriber recruitment.

### Ethics

The study received approval from the institutional review boards at the University of Wisconsin-Madison.

### Trial status

The recruitment phase of the trial is nearly completed and baseline data collection has begun.

## Discussion

Opioid misuse has become a leading cause of substance use disorder (SUD) admissions in the USA, second only to alcohol misuse [55]. Traditionally, the most frequent treatment for opioid disorders by specialty treatment organizations has been behavioral therapy steeped in the 12-step tradition of Alcoholics Anonymous, which many interpret as calling for an abstinence-only approach to treatment that precludes the use of any medications [56]. More recently, research has shown greater treatment retention rates and reduced use of illicit opioids with pharmacotherapy compared to behavioral therapy [3, 6, 57]. Hence, pharmacotherapy provides the most promising therapy to treat those wanting to reduce the adverse consequences of opioid misuse. The lack of reimbursement for OUD pharmacotherapy [58], philosophical resistance to pharmacotherapy to treat SUDs [59], and limited availability of buprenorphine prescribers inhibit use of buprenorphine in addiction treatment settings [60]. The most widely endorsed barrier among programs that do not use pharmacotherapy, however, is the lack of access to licensed prescribers [61]. Increasing OUD pharmacotherapy prescribing capacity is imperative in addressing the opioid epidemic.

This trial addresses a structural barrier to use of OUD pharmacotherapies, lack of OUD pharmacotherapy prescribing capacity. By increasing access to evidence-based OUD pharmacotherapies, this trial could have a significant public health impact by reducing the morbidity and mortality of opioid addiction. Should the PRB be successful, it would, to our knowledge, be the first evidence-based prescriber recruiting tool and could be used to recruit physicians and other prescribers to medically underserved areas or other areas where shortages exist.

## Abbreviations

ICC: Intraclass correlation
MAT: Medication-assisted treatment
NIATx: Network for the Improvement of Addiction Treatment
OUD: Opioid use disorder
PRB: Prescriber recruitment bundle
SUDs: Substance use disorders
